# Transcriptome and targeted metabolome analysis of lipid profiles, nutrients compositions and volatile compounds in longissimus dorsi of different pig breeds

**DOI:** 10.5713/ab.24.0564

**Published:** 2024-10-28

**Authors:** Zhen Luo, Ting Lai, Yijia Fan, Chengbin Yu, Wei Li, Meng Li, Shenghui Lei, Jing Zhang, Weina Xu, Zhe Wang, Jianxiong Xu

**Affiliations:** 1 Shanghai Key Laboratory of Veterinary Biotechnology/Shanghai Collaborative Innovation Center of Agri-Seeds, School of Agriculture and Biology, Shanghai Jiao Tong University, Shanghai, China; 2 Key Laboratory of Molecular Animal Nutrition, Ministry of Education, College of Animal Sciences, Zhejiang University, Hangzhou, China; 3 Jinan Laiwu Pig Industry Technology Research Institute Co., Ltd., Laiwu, China; 4 Shanghai Songjiang Center for Animal Disease Control and Prevention, Songjiang, China

**Keywords:** Intramuscular Fat, Lipid Profiles, Longissimus Dorsi, Pig Breeds, Volatile Compounds

## Abstract

**Objective:**

Improving meat quality is important for commercial production and breeding. The molecular mechanism of intramuscular fat (IMF) deposition and meat characteristics require further study.

**Methods:**

This study aimed to study the mechanism of IMF deposition and meat characteristics including redox potential, nutrients compositions and volatile compounds in longissimus dorsi (LD) by comparing with different pig breeds including Shanghai white (SW), Duroc×(Landrace Yorkshire) (DLY) and Laiwu (LW) pigs.

**Results:**

Results showed that the contents of IMF, triglyceride (TG), total cholesterol (TC), and redox potential parameters were lower, while the content of malondialdehyde (MDA) and activity of lactate dehydrogenase (LDH) were higher in LD of SW pigs compared with LW pigs (p<0.05). No differences were observed about these parameters between SW and DLY pigs. Also, the contents of medium-long chain fatty acids and γ-aminobutyric acid (GABA) were higher, while Asp was lower in LD of SW pigs compared with LW pigs (p<0.05). Volatile compounds results showed that 6 ketones, 4 alkenes, 11 alkanes, 2 aldehydes, 1 alcohol were increased, and cholesterol was decreased in SW pigs compared with LW pigs. Transcriptome results showed that differential expressed genes involved in lipid synthesis, metabolism and transport in LD between SW and LW pigs, which were further verified by quantitative polymerase chain reaction. Spearman correlation showed that HSL and Nedd4 were positively related to contents of TG and IMF, while negatively related to volatile compounds and fatty acids (p<0.05). Plin3 and Mgll were negatively related to contents of TG, IMF and cholesterol, while positively related to MDA, LDH, and volatile compounds (p<0.05). PPARA was negatively related to contents of TC and IMF, and activity of superoxide dismutase, while positively related to volatile compounds (p<0.05).

**Conclusion:**

Our study provided new insights into potential mechanisms of IMF deposition, nutrients composition and volatile compounds of muscular tissues of different pig breeds.

## INTRODUCTION

China is the world’s largest producer and consumer of pork [1]. With the improvement of living standards, better pork quality requirements are increasing except for pork quantity. Meat quality is the most important economic traits of pigs that include moisture, intramuscular fat (IMF), pH, meat color, water-holding capacity and tenderness, which is directly related to the economic benefits of pigs [2]. Pork contains valuable nutrients that are important for human health, including amino acids (AAs), nucleotides, vitamins, minerals and fatty acids, which also determines pork nutritional value and flavor, and influences consumer acceptance and purchase intent in pork marketing [3]. IMF, corresponding to the amount of fat within muscles, is positively related to meat quality including juiciness, tenderness, and flavor [4]. IMF content was variable and affected by many factors including breed, diet, and environment, among which the breed contributes the most [5,6]. Since IMF-related genes are enriched in lipid metabolism processes including fatty acid uptake, β-oxidation, lipid synthesis, storage and lipolysis, which also determines meat sensory and nutritional quality [5]. Thus, understanding the potential mechanism of lipid metabolism and meat characteristics, and seeking for solution to promote IMF deposition and improve meat quality are important.

China also has many local native breeds due to long-term natural and artificial selection, which have higher IMF contents and better meat characteristics (such as strong water-holding capacity, tender and juicy) than commercial pigs such as crossbreed Duroc×(Landrace Yorkshire) (DLY) pigs [7,8]. Among them, Laiwu (LW) pigs are famous for its tender meat, bright color, rich flavor with highest IMF content, representing an excellent local breed in North China [7]. Shanghai white (SW) pig, which was bred in the late 1970s, is a local composite breed mainly made up of Yorkshire pigs, Subai pigs and Taihu pigs crossbred in Shanghai. This breed is famous for strong adaptability, higher reproduction rate and higher carcass lean meat percentage [9]. However, until now, the molecular mechanism of IMF deposition among these pig breeds and meat characteristics including nutrient compositions especially in SW pigs are unknown. Therefore, the study aimed to investigate the lipid profiles, nutrient compositions and volatile compounds and potential mechanisms of IMF deposition in longissimus dorsi (LD) by comparing DLY, LW and SW pigs.

## MATERIALS AND METHODS

### Animal care

The present experiment was reviewed and approved by the ethics committee of Shanghai Jiao Tong University approved this study (202201188).

### Sampling and intramuscular fat analysis

The fresh LD of castrated male LW (Body weight: 115.875 ±7.817 kg, n = 8), SW (102.400±2.011 kg, n = 8) and DLY (107.250±2.340 kg, n = 8) kg pigs were obtained from Jinan Laiwu Pig Industry Technology Research Institute Co., Ltd (Laiwu, China), Shanghai Swine Testing Center in Songjiang District (Shanghai) and Chongming district (Shanghai), respectively. The average slaughter ages were 177.500±0.945 d (SW), 300.375±1.194 d (LW), 173.600±1.580 d (DLY) respectively, which had reached the standardized market slaughter age and weight. All the pigs were reared under standard ventilation conditions and free access to diets and water. The dietary ingredients and nutritional levels were listed in Supplement 1, 2. Samples were immediately placed on ice or stored in liquid nitrogen and transferred to the laboratory for analysis. The IMF content was analyzed in muscle tissues using the Soxhlet method with petroleum ether as the solvent and quantified as the weight percentage of wet muscle tissue.

### Morphological analysis

The fresh LD were also fixed in 4% paraformaldehyde and embedded in paraffin, sectioned and stained with hematoxylin and eosin (H&E) for morphological analysis. The muscle samples were also embedded in frozen liver section for oil red staining to visualized lipid droplets.

### Triglyceride, total cholesterol and lactate dehydrogenase determination

The muscle tissues were homogenized in phosphate-buffered saline buffer and the supernatants were gathered after centrifuging 3,000×g for 15 min. Protein concentration in the supernatants was measured through the bicinchoninic acid (BCA) protein assay kit. The content of triglyceride (TG), total cholesterol (TC) and activity of lactate dehydrogenase (LDH) in these samples were determined according to the manufacturers’ instructions (Nanjing Jiancheng Bioengineering Institute, Nanjing, China).

### Redox parameters determination

The redox parameters were determined according to the manufacturers’ instructions. Protein concentration was measured through BCA protein assay kit. Superoxide dismutase (SOD), malondialdehyde (MDA), GSH/GSSG (Nanjing Jiancheng Bioengineering Institute) and NAD^+^/NADH (Shanghai Beyotime Biotechnology, Shanghai, China) were measured and the absorbance at 450, 530, 405 and 450 nm was recorded, respectively.

### Transcriptome sequencing

The RNA of muscle tissues were extracted with the RNAiso Plus (#9108, TaKaRa, Kyoto, Japan). The results of RNA quality assessment were shown in Supplement 3. After mRNA purification and fragmentation, RNA was reverse transcribed into cDNA. Polymerase chain reaction (PCR) amplification was used for enrichment of library fragments, and 450 bp fragments were selected. Library quality was assessed on the Agilent 2100 Bioanalyzer. The library preparations were sequenced on an Illumina Hiseq platform and paired-end reads were generated. To ensure the RNA-seq data quality for subsequent analysis, the sequencing error rate distribution and A/T/G/C content distribution were checked, and low quality (average base mass value<20) and N>5 (undetermined base) were filtered out to obtain clean reads. Reads were mapped to the rat genome and were annotated. The differential expression analysis, enrichment analysis and clustering analysis of the samples were further performed.

### RNA isolation, cDNA synthesis and quantitative polymerase chain reaction

The total RNA from muscle tissues was extracted following the instructions of the RNA extraction kit. The concentration of RNA was quantified using a spectrophotometer (ThermoFisher Scientific, Chadds Ford, PA, USA). Then 1 μg of RNA was reverse transcribed into cDNA using the PrimeScrip RT reagent Kit. The real-time quantitative PCR reaction was applied to quantify the gene expression with the LightCycler96 system (Roche, Basel, Swiss). The reaction was performed in a total volume of 20 μL, including 10 μL SYBR Green mix, 2 μL cDNA, 7.2 μL H2O, and 0.4 μL each of forward and reverse primers. Amplification conditions were initial 95°C for 30 s, 40 cycles of 95°C for 5 s and 60 °C for 20 s, and a melting curve data. The primers were designed and listed in Supplement 4. The quantification of the genes in each sample was performed in triplicate. β-actin was used as a housekeeping gene to normalize target gene transcript levels. The relative expression of target genes can be calculated by comparing the CT values of target genes and β-actin. The values of SW and DLY pigs were expressed as a fold of the LW pigs, using the formula 2-(^ΔΔ^Ct), where ΔΔCt = (Ct _Target_–Ct _β-actin_) treatment–(Ct _Target_–Ct _β-actin_) model.

### Medium-long chain fatty acids determination

A targeted metabolomics approach was used to study the compositions of medium-long chain fatty acids in LD according to our previous study [10]. Briefly, 10% H_2_SO4-CH_3_OH solution (600 μL) were added into 50 mg sample, suspended for 1 min and bathed in water at 62°C for 2 h. After cooling, anhydrous sodium sulfate and 600 μL n-hexane were added and suspended for 1 min. Then, the solution was centrifuged at 3,500 r/min for 5 min. The supernatants were dried vacuum, dissolved in 200 μL n-hexane and analyzed by gas chromatograph/mass spectrometer (GC-MS). The samples were separated by gas chromatography on a DB-5MS capillary column (30 m×0.25 mm ID×0.25 μm). A standard sample mixture was used to identify the fatty acids profiles. The concentrations were calculated based on the chromatogram peak areas.

### Amino acids determination

A targeted metabolomics approach based on multiple reaction monitoring (MRM) was used to quantify the contents of AAs according to our previous study [10]. The sample pretreatment was the same to the UHPLC-QTOF-MS. The samples were separated by Agilent 1290 Infinity LC ultra-performance liquid chromatography. Mobile phase A were 25 mM ammonium formate+0.08% FA aqueous solution, and mobile phase B was 0.1% FA acetonitrile. The sample was placed in an automatic sampler at 4°C. The column temperature was 40°C and the flow rate was 250 μL/min. The injection volume was 2 μL. The gradient of liquid phase is as follows: mobile phase B decreases linearly from 90% to 70% during 0 to 12 min, decreases linearly from 70% to 50% during 12–18 min, decreases linearly from 50% to 40% during 18 to 25 min, increases linearly from 40% to 90% during 30 to 30.1 min and maintained at 90% during 30.1 to 37 min. A 5500 QTRAP mass spectrometer (AB SCIEX) was used for mass spectrometry analysis under positive ion mode. The conditions are as follows: source temperature 500°C, ion source gas1 (GAS1): 40, GAS2: 40, curtaingas: 30, ionsapary voltage floating: 5500 V. The ion pair was detected using the MRM mode. The contents of AAs were expressed as ng/mg in muscle tissue.

### Volatile compounds determination

Analyses of volatile compounds were performed on an Agilent 7890A gas chromatograph coupled with an Agilent 5975C series mass spectrometer (Agilent Technologies, Santa Clara, CA, USA). Briefly, one gram of samples were weighed in sample bottle. 3 mL of 1 mol/L sodium chloride solution were added, balanced at 60°C for 40 min and analyzed in GC-MS (Agilent Technologies). GC conditions were: inlet temperature 250°C, high purity helium, 1 mL/min carrier gas flow rate, 300°C transmission line temperature, DB-5MS column (30 m×0.25 mm×0.25°C). Heating procedure were: the initial temperature was 40°C and maintained for 1 min, then it was heated to 250°C at a speed of 5°C/min and maintained for 2 min. MS conditions were: 230°C ion source temperature, 70 eV ionization energy, 50 to 500 mz mass scanning range.

### Statistical analysis

The data in three groups were analyzed with one-way analysis of variance (ANOVA) followed by post-hoc Duncan’s test using the statistical software SPSS 17.0 (SPSS Inc., Chicago, IL, USA), while AAs data were analyzed with independent sample t test. Data were presented as mean±standard error of the mean. A p-value of <0.05 was considered statistically significant.

## RESULTS

### Intramuscular fat, lipid contents and redox parameters in longissimus dorsi of pigs

**Figure 1:**
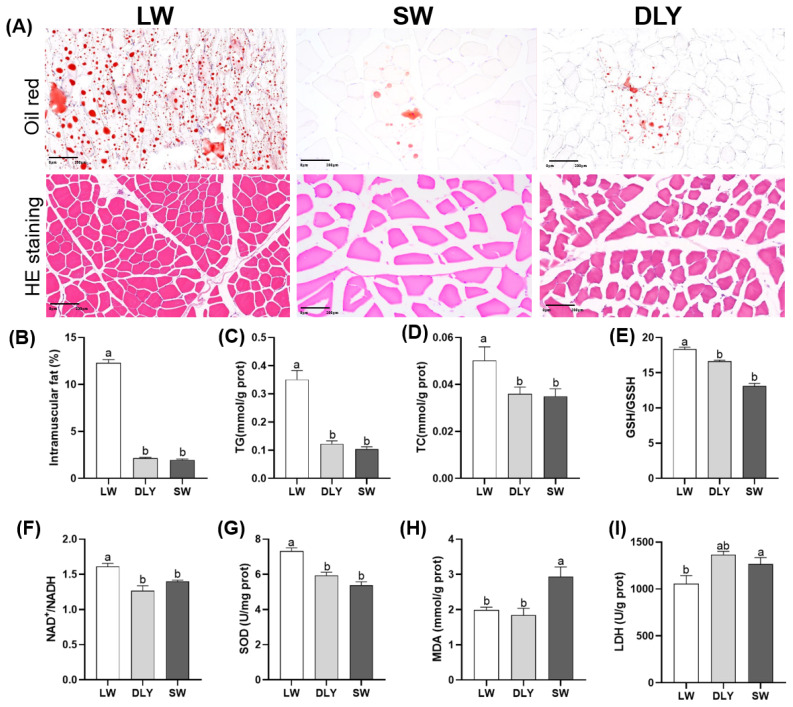
The morphology, and contents of IMF, TG and TC, and redox parameters in the LD of LW, DLY and SW pigs. (A) HE staining and oil red staining. (B) IMF, (C) TG, (D) TC, (E) SOD, (F) MDA, (G) LDH. ^a,b^ Different letters represent significant differences (p<0.05). IMF, intramuscular fat; TG, triglyceride; TC, total cholesterol; SOD, superoxide dismutase; MDA, malonaldehyde; LDH, lactic dehydrogenase; LW, Laiwu pigs; DLY, Duroc×(Landrace Yorkshire); SW, Shanghai white.

H&E staining showed that LD muscle fiber of LW pigs is closely arranged (Figure 1A). Muscle fiber diameter is small and muscle fiber density is high, while muscle fiber diameter and density in SW and DLY were large and low. Oil red staining results showed that the number of lipid droplets in LD of LW pigs were more than those in SW and DLY pigs (Figure 1A). IMF content (about 12%) was significantly higher in LD of LW pigs than that in SW and DLY pigs (p<0.05) (Figure 1B). No difference of IMF content was observed between SW and DLY pigs (p>0.05). The contents of TG and TC in LD of LW pigs were highest, followed by SW and DLY pigs. There were no significant difference of TG and TC between SW and DLY pigs (p>0.05) (Figures 1C, 1D).

The activity of SOD, ratios of GSH/GSSG and NAD^+^/NADH in LD of LW pigs were significantly higher (p<0.05) than those of SW and DLY pigs (Figures 1E–1G). The content of MDA in LD of LW and SW pigs was significantly lower than that of DLY pigs (Figure 1H). The activity of LDH in DLY pigs was significantly higher than that of LW pigs (Figure 1I). These results suggested that muscle tissues of LW pigs had better lipid deposition and higher antioxidant ability than SW and DLY pigs. No significant difference of lipid deposition and higher antioxidant ability were observed between SW and DLY pigs.

### Medium-long chain fatty acids contents

The contents of medium-long chain fatty acids including saturated fatty acids (SFA), monounsaturated fatty acids (MUFA) and polyunsaturated fatty acids (PUFA) in the LD of pigs were determined by targeted metabolomics (Table 1). Our results showed that total SFA, MUFA and PUFA were significantly higher (p<0.05) in SW pigs compared with LW and DLY pigs, while total SFA, MUFA and PUFA were not significantly different between LW and DLY pigs (p>0.05). SFA including pentadecanoic acid, palmitic acid, margaric acid, stearic acid, arachidic acid, heneicosanoic acid and tricosanoic acid, MUFA including myristelaidic acid, Oleic acid, cis-11-eicosenoic acid and erucic acid, and elaidic acid, PUFA including linoelaidic acid, γ-linolenic acid, eicosapentaenoate, arachidonic acid, cis-11,14,17-eicosatrienoic acid, docosapentaenoate (C22:5n-6), adrenic acid and 13C,16C-docosadienoic acid were higher in SW pigs compared with LW and DLY pigs (p<0.05). MUFA such as heptadecanoic acid was significantly lower in SW pigs when compared with LW and DLY pigs. The SFA including lauric acid, MUFA including trans-10-heptadecenoic acid (C17:1T) were significantly higher in SW pig compared with DLY pigs (p<0.05). MUFA such as trans-11-eicosenoic acid was significantly higher in SW pig compared with LW pigs (p<0.05).

### Amino acids and derivatives contents

The 8 essential amino acids (EAAs) including Lys, Met, Thr, Trp, Phe, Leu, Ile and Val were identified in the LD of two breeding pigs (Table 2). There were no difference of EAAs (p>0.05) in the LD between LW and SW pigs. The non-essential amino acids (NEAAs) including Asp, Tyr, Ser, Gly, Glu, Pro, Arg, Gln, Asn and His were identified in the LD of pigs except for Ala. The content of Asp was significantly lower (p<0.05) in SW pig compared with LW pigs. AAs derivative γ-aminobutyric acid (GABA) was significantly higher (p<0.05) in SW pigs compared with LW pigs, while ornithine (Orn) content was not different between these two pigs (p>0.05).

### Volatile compounds analysis

**Figure 2:**
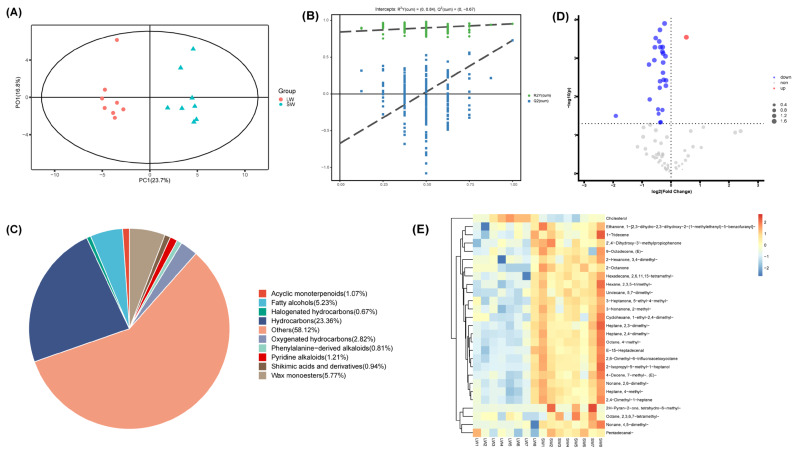
The differential volatile compounds of LD between LW and SW pigs determined by GC-MS. (A) OPLS-DA score plot. (B) OPLS-DA permutation test. (C) The classification of identified metabolites. (D) Volcano plot. (E) Hierarchical clustering of differential volatile compounds between LW and SW. LD, longissimus dorsi; LW, Laiwu pigs; SW, Shanghai white; GC-MS, gas chromatograph/mass spectrometer; OPLS-DA, orthogonal partial least squares discriminant analysis.

The differential volatile compounds of LW and SW samples were further studied. Orthogonal partial least squares discriminant analysis (OPLS-DA) results showed that these samples were divided into 2 clusters (Figure 2A). OPLS-DA with 7-fold cross-validation showed R2Y = 0.954 and Q2 = 0.728. There was no overfitting in permutation test (Figure 2B). Totally, 745 metabolites were ultimately identified, including hydrocarbons (15.962%), wax monoesters (5.77%), fatty alcohols (5.23%), oxygenated hydrocarbons (2.82%), pyridine alkaloids (1.21%), acyclic monoterpenoids (1.07%), shikimic acids and derivatives (0.94%), phenylalanine-derived alkaloids (0.81%), halogenated hydrocarbons (0.67%), and others (58.12%) (Figure 2C). A variable importance in projection score>1 and p<0.05 were used as criteria for differential metabolite screening. Totally, 27 differential metabolites were identified. Compared with SW pigs, 26 differential metabolites were decreased, and one metabolite (cholesterol) was increased in LW pigs (Figure 2D). The down-regulated compounds including ketones (2-Octanone, 2-methyl-3-Nonanone, 3,4-dimethyl-2-Hexanone, 5-ethyl-4-methyl-3-Heptanone, 1-[2,3-dihydro-2,3-dihydroxy-2-(1-methylethenyl)-5-benzofuranyl]-Ethanone, 2′,4′-Dihydroxy-3′-methylpropiophenone), alkenes (2,4-Dimethyl-1-heptene,7-methyl-(E)-4-Decene, (E)-9-Octadecene, 1-Tridecene), alkanes (4-methyl-Heptane, 2,4-dimethyl-Heptane, 2,3-dimethyl-Heptane, 2,3,5-trimethyl-Hexane, 1-ethyl-2,4-dimethyl-Cyclohexane, 4,5-dimethyl-Nonane, 2,6-dimethyl-Nonane, 4-methyl-Octane, 2,3,6,7-tetramethyl-Octane, 2,6,11,15-tetramethyl-Hexadecane, 5,7-dimethyl-Undecane), aldehydes (Pentadecanal-, and E-15-Heptadecenal), alcohol (2-Isopropyl-5-methyl-1-heptanol), and others (tetrahydro-6-methyl-2H-Pyran-2-one and 2,6-Dimethyl-6-trifluoroacetoxyoctane) (Figure 2E).

### Transcriptome analysis of differential expressed genes in LD

**Figure 3:**
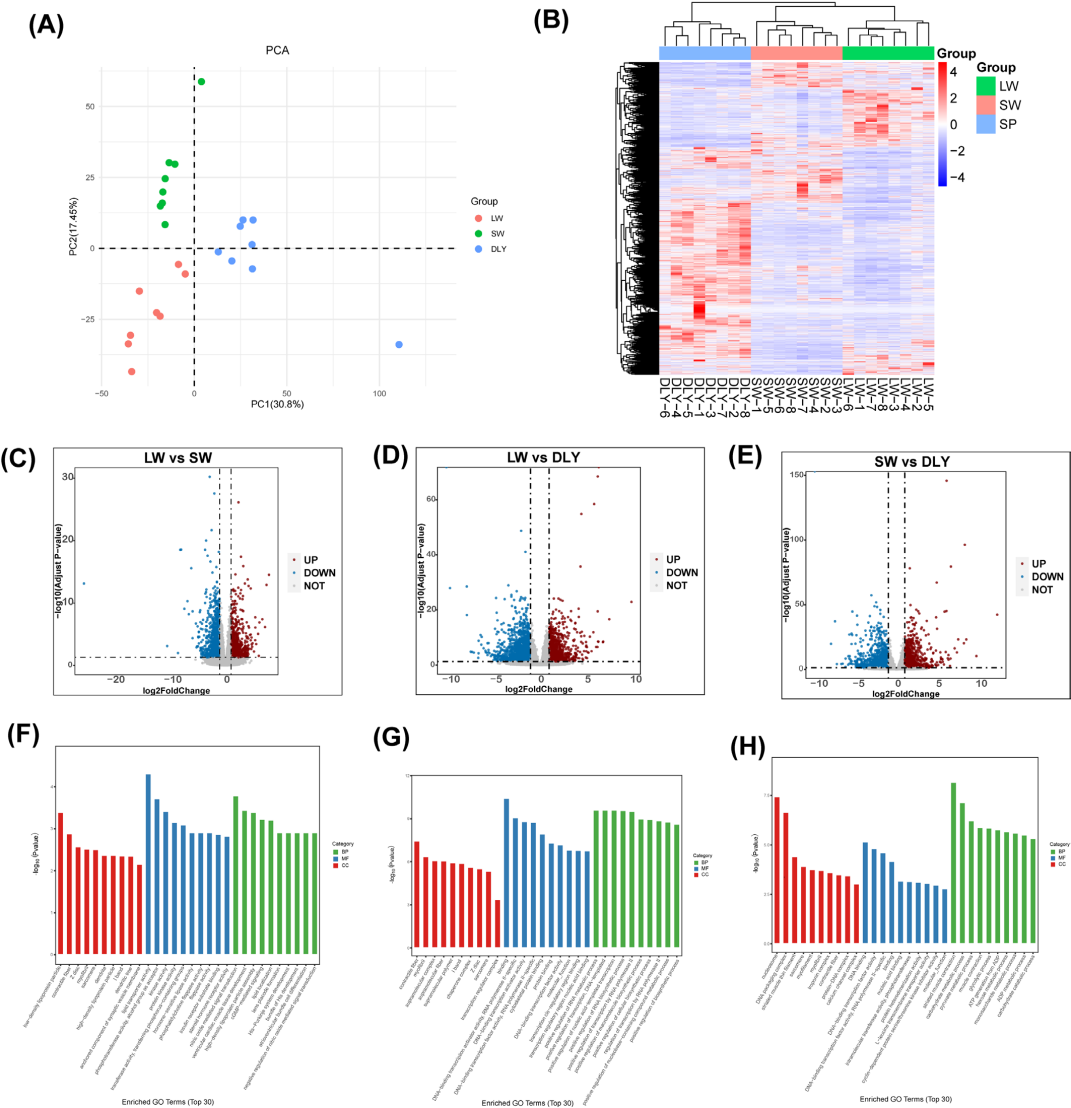
Transcriptome analysis of DEGs in LD of LW, DLY and SW pigs. (A) PCA. (B) DEGs cluster analysis. (C–E) Volcano plot of LW vs SW pigs, LW vs DLY pigs and SW vs DLY pigs. p<0.05 and |log2FC|>1 were used as the criterion for screening. (F–H) GO pathways enrichment of LW vs SW pigs, LW vs DLY pigs and SW vs DLY pigs. PCA, principal component analysis; LW, Laiwu pigs; SW, Shanghai white; DLY, Duroc×(Landrace Yorkshire); DEGs, differential expressed genes; GO, Gene Ontology.

The differential expressed genes (DEGs) in LD of LW, DLY and SW pigs were further analyzed by transcriptome. The samples of three groups were basically divided into three clusters by principal component analysis (PCA) (Figure 3A). p<0.05 and |log2FC|>1 were used as the criterion for screening. The DEGs cluster analysis and volcano plot among these pig breeds were shown in Figures 3B–3E. Totally, 2120 DEGs were down-regulated, and 939 genes were up-regulated in LD of LW pigs compared with DLY pigs. 1524 genes were down-regulated, and 931 genes were up-regulated in LD of SW pigs compared with DLY pigs. 801 genes were up-regulated, and 1074 genes were down-regulated in LW pigs compared with SW pigs. Compared with SW pigs, go enrichment analysis of LD from LW pigs showed that several genes focused on biological process including low-density lipoprotein particle, contractile fiber, high-density lipoprotein particle and so on (Figure 3F). Several genes focused on molecular function including lipid transporter activity, hormone-sensitive lipase activity, steroid hormone receptor activity and so on. A few genes focused on cellular component including ventricular cardiac muscle tissue development, high-density lipoprotein particle assembly, lipid localization and so on. Compared with DLY pigs, go enrichment analysis of LD from LW pigs showed that several genes focused on biological process including contractile fiber, myofibril, supramolecular fiber and so on (Figure 3G). A few genes focused on molecular function including binding, cytoskeletal protein binding and so on. A few genes focused on cellular component including positive regulation of RNA metabolic process, positive regulation of nucleic acid-templated transcription and so on. Compared with DLY pigs, go enrichment analysis of LD from SW pigs showed that a number of genes focused on biological process including nucleosome, DNA packaging complex and so on (Figure 3H). Several genes focused on molecular function including DNA binding, DNA binding transcription factor activity and so on. A number of genes focused on cellular component including striated muscle contraction, carbohydrate metabolic process, muscle contraction and so on. The results of Kyoto encyclopedia of genes and genomes (KEGG) enrichment analysis did not show related pathways among LW, DLY, and SW pigs.

### Quantitative polymerase chain reaction verification and correlation analysis

**Figure 4:**
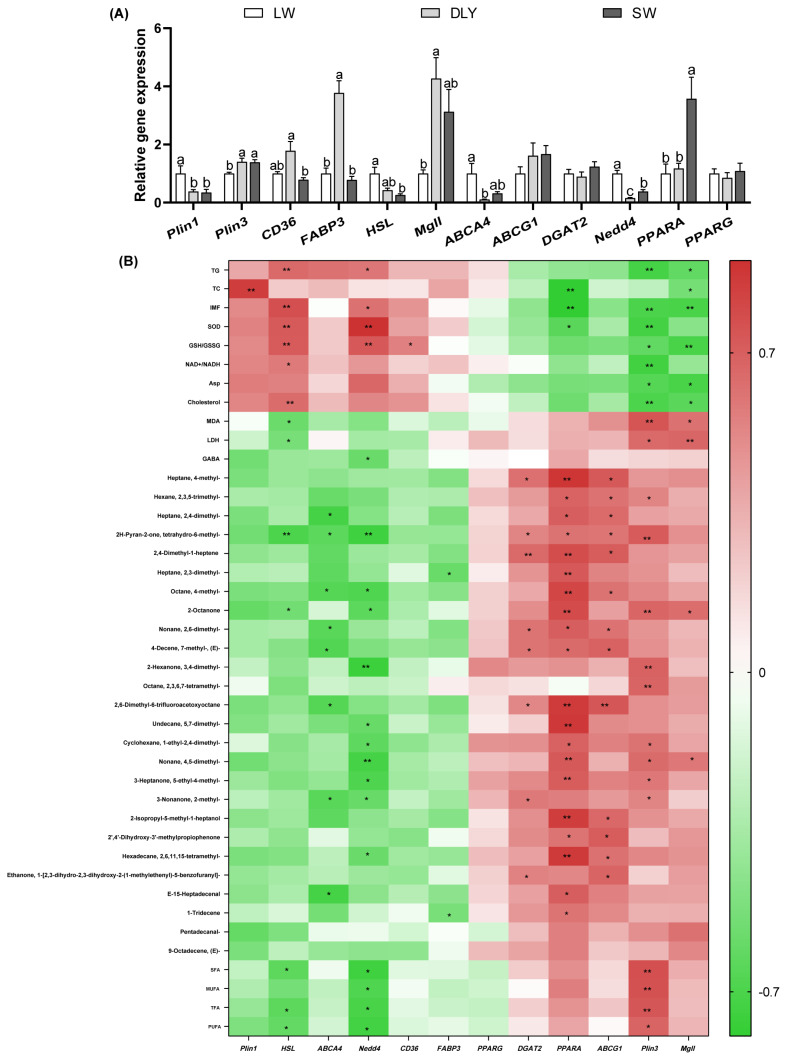
Gene expression was verified by qPCR and spearman correlation between nutrients compositions volatile compounds, redox parameters and gene expression in LD. (A) qPCR results. β-actin was used as a housekeeping gene to normalize target gene transcript levels. Different letters represent significant differences (p<0.05). (B) Spearman correlation results, * p<0.05, ** p<0.01. LW, Laiwu pigs; DLY, Duroc×(Landrace Yorkshire); SW, Shanghai white; TG, triglyceride; TC, total cholesterol; IMF, intramuscular fat; SOD, superoxide dismutase; MDA, malondialdehyde; GABA, γ-aminobutyric acid; SFA, saturated fatty acids; MUFA, monounsaturated fatty acids; TFA, trans fatty acids; PUFA, polyunsaturated fatty acids; qPCR, quantitative polymerase chain reaction; LD, longissimus dorsi.

The gene expression of lipid reserve, synthesis, transport and hydrolysis were further verified. Results showed that gene expression of *Plin1* was higher, but Plin3 was lower (p<0.05) in LW pigs than in LW and DLY pigs (Figure 4A). The gene expression of *CD36* and *FABP3* were significantly higher (p<0.05) in DLY pigs compare with SW pigs and there were no difference between LW and SW pigs (p>0.05). *Mgll* expression was significantly lower (p<0.05) in LW pigs compared with DLY pigs, and *Mgll* expression was not different (p>0.05) between LW and SW pigs. *HSL* expression was higher in LW pigs compared with SW pigs (p<0.05) and not different between DLY and SW pigs (p<0.05). *ABCA4* and *Nedd4* expression were significantly higher in LW pigs than in DLY pigs (p<0.05) (Figure 4B). PPARA expression was lower in LW and DLY pigs than in SW pigs (p<0.05). There were no difference of *ABCG1*, *DGAT2* and *PPARG* expression among these three pigs (p>0.05).

Furthermore, the spearman correlation between the biochemical index (including lipid contents, redox parameters, fatty acids, AAs and volatile compounds) and gene expression in LD was analyzed (Figure 4B). The expression of *Plin1* was positively (p<0.05) related to contents of TC. The expression of *HSL* and *Nedd4* were positively related to contents of TG, IMF, GSG/GSSG, and activity of SOD, while negatively related to 2H-Pyran-2-one, tetrahydro-6-methyl-, 2-Octanone, SFA, trans fatty acids (TFA) and PUFA (p<0.05). The expression of Plin3 and *Mgll* were negatively (p<0.05) related to contents of TG, IMF, GSG/GSSG, cholesterol and Asp, while positively (p<0.05) related to MDA, LDH, 2-Octanone, and Nonane, 4,5-dimethyl-(p<0.05). Also, the expression of Plin3 was positively related to SFA, MUFA, TFA, PUFA and many volatile compounds such as Hexane, 2,3,5-trimethyl-, 2-Octanone and so on (p<0.05). The expression of PPARA was negatively (p<0.05) related to contents of TC and IMF, and activity of SOD, while positively related to many volatile compounds such as heptane, 4-methyl-, 2H-Pyran-2-one, tetrahydro-6-methyl- and so on, which were similar to *ABCG1* expression (p<0.05).

## DISCUSSION

MDA is the end product of lipid peroxidation, which could decrease meat quality by increasing of discoloration, decreasing of water-holding capacity and nutritive values, and production of toxic compounds and off-flavor [11]. The ratio of GSH/GSSG and NAD^+^/NADH are important indicator of cellular health and redox potential, which protect meat against oxidative deterioration and are used as potential biomarkers to predict meat quality [12,13]. Previous study reported that diet supplementation with NAD^+^ precursor nicotinic acid improved carcass characteristics by increasing IMF content, marbling score, redness (a*) and chroma (C*) values of LD muscle of steers [14]. In this study, the higher ratio of GSH/GSSG and NAD^+^/NADH, increased SOD activity and lower MDA in LD of LW pigs suggested higher intrinsic antioxidant defense systems and stress resistance of LW pigs than SW pigs. LDH is involved in glycolysis and its activity is high in glycolytic muscle fibers. The lower LDH activity indicated less glycolytic myofibers in LD of LW pigs compared with SW pigs. Similarly, LDH activity was reported to be lower in M. longissimus of Chinese local Bama pigs compared with Landrace pigs [15]. Thus, the specific muscle fiber parameters such as muscle fiber size and area in these pig breeds need future study.

IMF (marbling) is an important factor determining meat tenderness, color, flavor and nutritional value. Fatty acids including SFA, MUFA and PUFA play an important role in IMF deposition and meats characteristics. The most abundant fatty acids in our results were palmitic acid, oleic acid, α-linolenic acid and elaidic acid. SFA such as palmitic acid appeared more powerful at inducing IMF deposition in pigs [16]. The content of SFA are positive correlations with oxidative stability, fat firmness and IMF content, and negatively correlated with drip loss [17]. In this study, SFA such as palmitic acid was increased in SW pigs compared with LW and DLY pigs, which is inconsistent with IMF content and needs further investigation. Trans fatty acids (TFA), belong to unsaturated fatty acids, its content in pigs’ tissues is lower and is positively proportional to long-term dietary intake [18]. The lipogenic effect of TFA such as increased levels of LDL cholesterol are more than unsaturated fats, and as much as SFA [19]. In this study, the contents of elaidic acid and linoelaidic acid (the trans geometric isomer of oleic and linoleic acids) in SW pigs were higher than those in LW and DLY, which is contrary to IMF content, suggesting that diet is not the major factor in inducing IMF deposition in different pig breeds. Nevertheless, the diets of pigs were different, which is one of the potential limitations in this study. PUFA are structural components of cell membrane that are essential for life. The effects of PUFA on meat quality of pigs such as IMF content are inconsistent. In a meta-analysis, PUFA supplementation could improve the meat quality of pigs by increasing IMF content [20]. Recently, PUFA contents were positively related to the values of L* and b* and inversely related to IMF content in muscle of different breeds [21,22]. In our study, PUFA such as γ-linolenic acid, α-linolenic acid, arachidonic acid and docosapentaenoate (C22:5n-6) were higher in SW pigs than those in LW and DLY pigs. Linoleic acid metabolic pathways and arachidonic acid were negatively correlated with IMF deposition in muscle tissue [23]. Docosapentaenoate (C22:5n-6) is an intermediate product between eicosapentaenoic acid and docosapentaenoic acid that exhibit good efficacy in lowering diet-induced lipids [24]. All these studies suggested LD of SW pigs has abundant PUFA with high nutritional value, which was negatively correlated with IMF content.

AAs contents in the muscle not only contribute to nutritional value, but also affect meat quality such as flavor. In this study, compared with LW pigs, Asp was lower while GABA was higher in LD of SW pigs. Asp, belongs to the umami AAs, is significantly associated with meat color (a* values, redness), which is a very important meat quality trait and directly affect consumer acceptance and purchase intent [25]. The increased Asp in the LW pigs probably explained consumer preference for different types of pork. GABA is produced through α-decarboxylation of glutamic acid catalyzed by glutamate decarboxylase, which has beneficial effects on promoting daily gain, improving antioxidant status and decreasing inflammatory response in animals [26]. GABA was reported to improve meat quality such as decreasing of drip loss and increasing of pH_45_min in longissimus muscle of growing-finishing pigs after transportation stress [27]. GABA content in cattle meat was negatively correlated with IMF content [28], which is similar to our study in pigs. These studies suggested GABA negatively regulated lipid deposition in muscular tissues of domestic animals. However, the potential molecular mechanism of GABA-mediated lipid deposition is unknown and needs further investigation.

**Figure 5:**
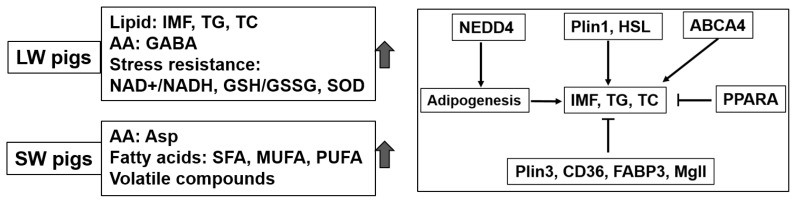
A schematic diagram summarizing the main results and mechanism of in IMF deposition between different pig breeds. LW, Laiwu pigs; IMF, intramuscular fat; TG, triglyceride; TC, total cholesterol; AA, amino acid; GABA, γ-aminobutyric acid; SOD, superoxide dismutase; SW, Shanghai white; SFA, saturated fatty acids; MUFA, monounsaturated fatty acids; PUFA, polyunsaturated fatty acids.

Perilipins (Plins) are abundant lipid droplet proteins that regulate lipid stores and hydrolysis. *Plin1* was increased during adipocyte differentiation and was positively related to IMF content in pigs [29]. *Nedd4* is a novel regulator of adipogenesis and cholesterol metabolism since knockdown of *Nedd4* in 3T3-L1 adipocytes suppressed adipocyte conversion [30]. In this study, the increased *Plin1* and *Nedd4* expression in LW pigs compared with DLY and SW pigs. Furthermore, *Plin1* expression was positively related to TC, and *Nedd4* expression was positively related to IMF and TG contents, suggested higher expression of *Plin1* and *Nedd4* are responsible for intramuscular lipid deposition, adipocyte conversion and adipogenesis of pigs. Contrarily, Plin3 expression decreased in LW pigs compared with DLY and SW pigs, and its expression is negatively to TC, IMF and TG contents. Plin3 is a small/nascent lipid droplet marker that widely expressed in tissue, while knockdown of Plin3 reduced lipid oxidation in myotubes from lean humans [31]. Thus, the specific role of Plin3 in lipid hydrolysis or oxidation in LD of pigs need further verification. *HSL* and *Mgll* are responsible for lipid catabolism by hydrolyzing DAG into monoacylglycerols (MAGs), and hydrolyzing MAG into glycerol and free fatty acids, respectively [10]. In this study, *Mgll* was lower and *HSL* was higher in LW pigs when compared with DLY pigs. *Mgll* expression was negatively related to contents of TG, TC and IMF, while *HSL* was positively related to contents of TG and IMF. This was similar to previous studies which showed that *HSL* was positively correlated with IMF, while *Mgll* was negatively correlated with IMF [32]. Of note, *HSL* was a candidate gene involved in fat deposition, which could directly interact with *Plin1* to form a complex on lipid droplets to regulate lipolysis and fatty acid biosynthesis [33]. The increased *HSL* expression was consistent with increased *Plin1* expression in LW pigs compared with SW pigs in our study, suggesting *Plin1*/*HSL* pathways play important role in lipid deposition of pigs. Fatty acid transporting systems (including *CD36* and *FABP3*) are required for uptake and utilization of long-chain fatty acids, which are highly expressed in cardiac and skeletal muscle tissues [34]. The ATP-binding cassette transporter *ABCG1* is responsible for export of cholesterol, phospholipids and oxysterols, while *ABCA4* is a transporter for phosphati-dylethanolamine in photoreceptor cells [35]. In this study, *ABCA4* expression was higher and *FABP3* was lower in LW pigs when compared with SW pigs and DLY pigs respectively, indicating different substrates transportation ability of LD from different pig breeds. PPARA, belong to a group of nuclear regulatory factors family, plays a key role in fatty acid catabolism by transcriptional regulation of genes involved in fatty acid oxidation. In this study, PPARA expression was negatively related to contents of TC and IMF, suggesting an important target that negatively regulating lipid deposition of pigs. All the above results indicate that differences in IMF deposition between breeds is probably the combined results of network regulation of lipid metabolism genes (Figure 5). Further studies are needed to explore the network regulation pathways based on the methods of integrative and systems biology.

Flavor is a very important attribute contributing to meat sensory quality and consumer choice. A variety of volatile organic compounds such as alkanes and alkenes, alcohols, aldehydes, ketones, esters, amides, and terpenes are responsible for meat flavor. Many factors have been reported to affect meat flavor including species, age, diet, muscle anatomical location and cooking methods [36]. In this study, 6 ketones, 4 alkenes, 11 alkanes, 2 aldehydes, 1 alcohol were decreased in LW pigs when compared with SW pigs. Ketones, as products of lipid oxidation, have fruity and creamy aromas. Alkanes and alkenes could be produced by radiolytic degradation of unsaturated fatty acids and AAs [37]. Aldehydes are produced by lipid degradation and Strecker AA degradation, which are associated with pleasant odors (fatty and fruity). Alcohols produced from degradation of linoleic acid, contribute less to the flavor than aldehydes [38]. In this study, increased volatile compounds in SW pigs indicated higher fatty, fruity and creamy aromas than LW pigs, which possibly correlated with consumers’ acceptance. Indeed, appropriate IMF increased flavor, juiciness and texture, and reduced off-flavor, while excessive IMF decreased volatile and taste compounds [39]. Dietary nutrients supplementation such as l-arginine also increased the contents of fatty acid and volatile compounds such as ketones in muscle from pigs, and increased tenderness, juiciness and overall liking score [40]. The increased volatile compounds in SW pigs are in accordance with the results of increased fatty acids and decreased redox potential, suggesting that differences in flavor compounds were primarily related to variance of precursors and the extent of lipid peroxidation. Thus, further studies are needed to explore the sensory analysis and its relationship with volatile compound profiles.

## CONCLUSION

This study reported that SW pigs have lower IMF, lipid contents and redox potential, but higher lipid peroxidation, fatty acids profiles (including SFA, TFA and PUFA), GABA and flavor volatile compounds (including 6 ketones, 4 alkenes, 11 alkanes, 2 aldehydes, 1 alcohol) in LD when compared with LW pigs. DEGs involved in lipid synthesis, metabolism and transporter between SW and LW pigs were verified. Furthermore, spearman correlation showed that the expression of *HSL* and *Nedd4* were positively related to contents of TG, IMF, GSG/GSSG, and activity of SOD, while negatively related to volatile compounds and fatty acids. The expression of PPARA, Plin3 and *Mgll* were negatively related to contents of IMF, while positively related to volatile compounds. Our study provided fundamental data about meat characteristics and nutrients compositions of muscular tissues from different pig breeds and genetic selection for high meat quality. Further studies are needed to explore the potential mechanism of these DEGs regulating IMF deposition and flavor, and breed high-quality and high-nutritional value of pork based on genetic engineering methods.

## Figures and Tables

**Table 1:** The contents of medium-long chain fatty acids profiles (ng/g tissue) in LD of three pig breeds

Item		LW	SW	DLY	p-value
SFA		736.354±93.251^b^	1455.182±183.671^a^	844.550±102.228^b^	0.002
Caproic acid	C6:0	1.240±0.017	1.229±0.004	1.212±0.003	0.196
Octanoic acid	C8:0	1.089±0.101	1.126±0.010	1.106±0.006	0.927
Decanoic acid	C10:0	1.085±0.051^b^	1.413±0.080^a^	1.31±0.084^a^	0.015
Hendecanoic acid	C11:0	1.657±0.005	1.668±0.005	1.657±0.004	0.207
Lauric acid	C12:0	4.800±0.348^ab^	5.885±0.475^a^	3.988±0.457^b^	0.023
Tridecanoic acid	C13:0	2.156±0.006^b^	2.180±0.007^a^	2.167±0.005^ab^	0.035
Myristic acid	C14:0	26.834±3.078	28.522±3.460	25.742±3.018	0.837
Pentadecanoic acid	C15:0	3.688±0.160^b^	6.248±0.529^a^	4.472±0.218^b^	0.000
Palmitic acid	C16:0	635.026±87.190^b^	1323.679±175.053^a^	749.893±96.956^b^	0.002
Margaric acid	C17:0	9.304±0.523^c^	18.083±1.970^a^	13.376±0.931^b^	0.000
Stearic acid	C18:0	6.507±0.274^b^	9.198±0.519^a^	4.975±0.175^c^	0.000
Arachidic acid	C20:0	11.276±0.842^b^	16.944±1.276^a^	8.860±0.500^b^	0.000
Heneicosanoic acid	C21:0	4.931±0.043^b^	5.647±0.338^a^	4.804±0.033^b^	0.005
Docosanoic acid	C22:0	8.102±0.624^ab^	9.956±0.814^a^	6.798±0.532^b^	0.012
Tricosanoic acid	C23:0	9.121±0.400^b^	13.031±1.120^a^	6.933±0.348^c^	0.000
Lignoceric acid	C24:0	9.537±0.846^ab^	10.373±0.988^a^	7.255±0.591^b^	0.034
MUFA		533.436±63.858^b^	1215.053±242.827^a^	532.601±51.990^b^	0.002
Myristelaidic acid	C14:1	7.166±0.249^b^	10.071±0.791^a^	5.887±0.216^c^	0.000
10-Pentadecenoic acid	C15:1	6.991±0.201^a^	14.441±3.065^ab^	5.475±0.169^b^	0.001
Palmitoleic acid	C16:1	19.775±1.553^a^	44.039±7.982^a^	11.109±0.915^b^	0.000
Heptadecanoic acid (cis-10)	C17:1	12.826±2.269^a^	3.756±0.185^b^	10.749±0.784^a^	0.002
Oleic acid	C18:1	36.776±4.445^b^	350.609±145.743^a^	27.003±3.337^b^	0.007
Cis-11-eicosenoic acid	C20:1	7.246±0.329^b^	9.742±0.737^a^	5.859±0.218^c^	0.000
Erucic acid	C22:1	29.471±5.987^b^	95.497±15.92^a^	6.377±0.295^c^	0.000
Nervonic acid	C24:1	11.511±0.735^b^	7.344±0.643^a^	19.895±2.365^c^	0.000
Hexadecanoic acid (trans-9)	C16:1T	47.118±6.463^ab^	56.996±9.349^a^	30.587±2.783^b^	0.026
Trans-10-eeptadecenoic acid (C17:1T)	C17:1T	9.820±0.626^ab^	17.437±2.579^a^	8.436±0.271^b^	0.000
Elaidic acid	C18:1T	327.149±46.693^b^	570.946±73.624^a^	389.351±47.896^b^	0.019
Trans-10-nonadecenoic acid	C19:1T	3.980±0.118^a^	5.291±0.649^ab^	3.521±0.105^b^	0.004
Trans-11-eicosenoic acid	C20:1T	6.016±0.586^b^	8.202±0.655^a^	6.540±0.556^ab^	0.058
Brassidic acid	C22:1T	7.591±2.279	8.13±3.104	5.361±0.412	0.607
PUFA		1,703.997±212.716^b^	3,117.922±372.312^a^	1,842.403±223.188^b^	0.003
Linoelaidic acid	C18:2TT	121.614±35.542^b^	563.422±106.87^a^	100.315±36.502^b^	0.000
γ-Linolenic acid	C18:3	11.211±0.386^b^	19.36±0.974^a^	11.418±0.6^b^	0.000
α-Linolenic acid	C18:3	1,191.954±135.571^b^	1,816.186±190.43^a^	1,367.435±159.267^ab^	0.044
Eicosapentaenoate	C20:5	241.627±35.954^b^	473.191±59.512^a^	223.757±28.004^b^	0.001
Arachidonic acid	C20:4	15.26±1.555^b^	30.837±1.885^a^	18.281±1.646^b^	0.000
Cis-11,14,17-eicosatrienoic acid	C20:3	44.248±4.954^b^	70.226±4.685^a^	39.175±3.292^b^	0.000
11C,14C-eicosadienoic acid	C20:2	16.962±1.656	18.714±1.511	20.505±1.621	0.298
Docosapentaenoate (C22:5n-6)	C22:5n-6	6.475±0.313^b^	12.215±1.093^a^	7.411±0.395^b^	0.000
Docosapentaenoate (C22:5n-3)	C22:5n-3	12.912±0.985^a^	15.376±1.280^a^	7.696±0.523^b^	0.000
Adrenic acid	C22:4	34.132±5.520^b^	84.68±10.086^a^	39.585±4.242^b^	0.000
13C,16C-docosadienoic acid	C22:2	7.602±1.279^b^	13.715±2.236^a^	6.825±1.523^b^	0.021

a–c Different letters in a row represent significant differences (p<0.05).

LD, longissimus dorsi; LW: Laiwu pigs; SW, Shanghai white; DLY, Duroc×(Landrace Yorkshire); SFA, saturated fatty acids; MUFA, monounsaturated fatty acids; PUFA, polyunsaturated fatty acids.

**Table 2:** AAs contents and derivatives in LD between SW and LW pigs (ng/mg sample)

Item	SW	LW
EAAs		
Lys	5,377.95±646.289	7,882.1±1,884.566
Met	2,693.85±260.836	3,206.9±525.991
Thr	2,172.1±297.246	2,516.15±395.102
Ile	2,194.05±217.525	2,423.4±302.621
Val	3,699±353.02	4,646.5±434.716
Leu	1,335.85±159.596	1,472.25±170.192
Phe	4,374.6±278.962	4,750.3±611.334
Trp	596.285±91.417	838.65±110.23
NEAAs		
Asp	353.273±70.928^b^	2,797.829±674.284^a^
Tyr	3865.35±381.561	3781.3±626.909
Ser	4,593.25±661.898	5,948.2±1,018.224
Gly	8,608±772.403	10,703.85±1,602.848
Gln	1,5815.33±119.487	23,066.86±4,957.294
Glu	6,348.45±760.015	5,058.95±1,026.316
Pro	2,330.55±289.261	2,942±425.307
Arg	8,434±1,188.585	15,020±4,087.24
Asn	1,712.95±192.593	2,125.25±364.359
Derivatives		
GABA	82.291±12.264^a^	44.402±11.465^b^
Orn	845.05±140.993	610.39±110.717
His	3,167.15±324.258	4,340.971±542.187

a,b Different letters represent significant differences (p<0.05).

AA, amino acids; LD, longissimus dorsi; SW, Shanghai white; LW: Laiwu pigs; EAAs, essential amino acids; Lys, lysine; Met, methionine; Thr, threonine; Ile, isoleucine; Val, valine; Leu, leucine; Phe, phenylalanine; Trp, tryptophan; NEAAs, non-essential amino acids; Asp, aspartic acid; Tyr, tyrosine; Ser, serine; Gly, glycine; Gln, glutamine; Glu, glutamic acid; Pro, proline; Arg, arginine; Asn, asparagine; GABA, γ-aminobutyric acid; Orn, ornithine; His, histidine.

## SUPPLEMENTARY MATERIAL

**Supplement 1.** The dietary nutrition levels of LW pigs

**Supplement 2.** The dietary ingredients and nutritional levels of SW pigs

**Supplement 3.** Results of RNA quality assessment

**Supplement 4.** Primers used in this study.
