# Supplementary material for: Transcriptome and targeted metabolome analysis of lipid profiles, nutrients compositions and volatile compounds in longissimus dorsi of different pig breeds
Source: Anim Biosci. 2024 Oct 28;38(5):1053–66. doi: 10.5713/ab.24.0564 (PMC12062803; doi:10.5713/ab.24.0564)
Supplement: Supplementary file 1 [file ab-24-0564-Supplementary-1.pdf]

Supplement 1. The dietary nutrition levels of LW pigs

	15-30kg	30-75kg	75-100kg
ME (MJ/kg)	13.89	12.47	14.03
CP (%)	15	12.5	11
Ca (%)	0.6	0.52	0.48
P (%)	0.5	0.42	0.38
Available P (%)	0.25	0.18	0.16
