# Supplementary material for: Transcriptome and targeted metabolome analysis of lipid profiles, nutrients compositions and volatile compounds in longissimus dorsi of different pig breeds
Source: Anim Biosci. 2024 Oct 28;38(5):1053–66. doi: 10.5713/ab.24.0564 (PMC12062803; doi:10.5713/ab.24.0564)
Supplement: Supplementary file 2 [file ab-24-0564-Supplementary-2.pdf]

Supplement 2 The dietary ingredients and nutritional levels of SW pigs

Ingredients	20-50kg	50-80kg	80-120kg
Corn	52	58.5	57.3
Flour	18	12.5	12.5
Bran		3.5	7.5
Fermented distiller's	4	6	6
grains Bread flour	6		
Soybean oil	0.5		
Soybean meal	14.5	14.5	11.7
Base mix 2	5		
Base mix 3		5	
Base mix 4			5
Total	100	100	100
CP (%)	15.5	14.998	14
EE (%)	3.058	2.649	2.699
CF (%)	3.965	4.429	4.645
Ash (%)	5.417	5.391	5.388
Ca (%)	0.59	0.619	0.568
P (%)	0.462	0.35	0.332
Net Energy (Kcal)	2401.369	2352.754	2322.978
Lys (%)	1.061	1.014	0.917
SID Lys-s (%)	0.952	0.901	0.803
