# Supplementary material for: Transcriptome and targeted metabolome analysis of lipid profiles, nutrients compositions and volatile compounds in longissimus dorsi of different pig breeds
Source: Anim Biosci. 2024 Oct 28;38(5):1053–66. doi: 10.5713/ab.24.0564 (PMC12062803; doi:10.5713/ab.24.0564)
Supplement: Supplementary file 3 [file ab-24-0564-Supplementary-3.pdf]

Supplement 3 Results of RNA quality assessment

Sample	RNA concentration (ng/μl)	volume(μl)	Total (ng)	RIN value	Level	Residual fetch times
SW-1	575	80	46	8.3	B	>1
SW-2	312	80	24.96	8.1	B	>1
SW-3	408	80	32.64	7.8	B	>1
SW-4	470	80	37.6	7.9	B	>1
SW-5	395	80	31.6	8.2	B	>1
SW-6	439	80	35.12	7.9	B	>1
SW-7	426	80	34.08	8	B	>1
SW-8	482	80	38.56	6.4	B	>1
DLY-1	350	80	28	7.7	B	>1
DLY-2	419	80	33.52	7.8	B	>1
DLY-3	360	80	28.8	8	B	>1
DLY-4	467	80	37.36	8	B	>1
DLY-5	426	80	34.08	8.4	B	>1
DLY-6	329	80	26.32	8.6	B	>1
DLY-7	357	80	28.56	8.7	B	>1
DLY-8	297	80	23.76	7.9	B	>1
LW-1	309	80	24.72	7.9	B	>1
LW-2	431	80	34.48	8.2	B	>1
LW-3	348	80	27.84	8.1	B	>1
LW-4	318	80	25.44	8.2	B	>1
LW-5	383	80	30.64	8.3	B	>1
LW-6	239	80	19.12	8.4	B	>1
LW-7	214	80	17.12	8.4	B	>1
LW-8	268	80	21.44	8.5	B	>1

Level B: The sample quality meets the requirements of sequencing for database construction, and the total amount meets the requirements of database construction once but less than twice, or the sample is slightly degraded but meets the requirements of database construction.
