# Supplementary material for: Transcriptome and targeted metabolome analysis of lipid profiles, nutrients compositions and volatile compounds in longissimus dorsi of different pig breeds
Source: Anim Biosci. 2024 Oct 28;38(5):1053–66. doi: 10.5713/ab.24.0564 (PMC12062803; doi:10.5713/ab.24.0564)
Supplement: Supplementary file 4 [file ab-24-0564-Supplementary-4.pdf]

Supplement 4 Primers used in this study.

Target	Primer sequence	No.
CD36	F: 5'- TAACCCAGGACCCTGAGACC-3’	NM_001044622.1
	R: 3'- CTGCCACAGCCAGATTGAGA-5'	
Plin1	F: 5'- CAACAAGGGCCTGACTTTGC-3’	NM_001038638.1
	R: 3'- ATTGCATACAGACGCCACCA-5'	
Plin3	F: 5'- CAAGCACTCAGCCTGATGGA-3’	NM_001031778.1
	R: 3'- GACTCAACCTGCTCTGGCTT-5'	
Mgll	F: 5'- ACTTCCAGGTGTTCGTCAGG-3’	NM_001143718.1
	R: 3'- TGTTGCAGACTCAGGACTGG-5'	
HSL	F: 5'- ACCTGACACTGCATGACCTG-3’	NM_214315.3
	R: 3'- GGTGCTAATCTCGTCTCGGG-5'	
FABP3	F: 5'- TTGTGACACTGGATGGAGGC-3’	NM_001099931.1
	R: 3'- TAAGTGCGAGTGCAAACTGC-5'	
PPARA	F: 5'- TCAGTCCATTGGTGAGGACA-3’	NM_001044526.1
	R: 3'- GGCATGAACTCCGTAGTGGT-5'	
PPARG	F: 5'- GCCCTTCACCACTGTTGATT-3’	NM_214379.1
	R: 3'- GAGTTGGAAGGCTCTTCGTG-5'	
Nedd4	F: 5'- CGGCAGATCTCTGAGGAAAC-3’	XM_021094888.1
	R: 3'- TGCTGGAATGATTTGAGCTG-5'	
ABCA4	F: 5'- CCTACCTGCAGGACATGGTT-3’	XM_021090184.1
	R: 3'- CGCAACTCCTTCTCCAAGAC-5'	
DGAT2	F: 5'- AAAGAATGGGAGTGGCAATG-3’	NM_001160080.1
	R: 3'- TTCTCCCCGAAGGAGTAGGT-5'	
ABCG1	F: 5'- AAAGAATGGGAGTGGCAATG-3’	XM_021071021.1
	R: 3'- TTCTCCCCGAAGGAGTAGGT-5'	
β-actin	F: 5'- CTGCGGCATCCACGAAACT-3’ R:	XM_003357928.4
	3'- AGGGCCGTGATCTCCTTCTG-5'	

CD36: Fatty acid translocase; *Plin1*: Perilipin 1; *Mgll*: Monoglyceride lipase; *HSL*: Hormone sensitive lipase; *FABP3*: Fatty acid-binding protein 3; *PPARA*: Peroxisome proliferator activated receptor alpha; *PPARG*: peroxisome proliferator activated receptor gamma; *Nedd4*: Nedd4 E3 ubiquitin protein ligase; *ABCA4*: ATP binding cassette subfamily A member 4; *DGAT2*: Diacylglycerol O-acyltransferase 2; *ABCG1*: ATP binding cassette subfamily G member 1.
